# Point of Care Ultrasound Identification of Multiple Rib Fractures in a Pediatric Patient with Osteogenesis Imperfecta Type 3

**DOI:** 10.3390/children9060864

**Published:** 2022-06-10

**Authors:** Samuel Enci Quek, Vigil James, Leodivica Castillo, Ronald Ming Ren Tan, Gene Yong-Kwang Ong

**Affiliations:** 1 Department of Emergency Medicine, KK Women’s and Children’s Hospital, 100 Bukit Timah Road, Singapore 229899, Singapore; samuel.quek@mohh.com.sg (S.E.Q.); vigiljames@gmail.com (V.J.); leodivica.castillo@kkh.com.sg (L.C.); ronald.tan.m.r@singhealth.com.sg (R.M.R.T.); 2 Duke-National University of Singapore Medical School, 8 College Road, Singapore 169857, Singapore

**Keywords:** pediatrics, osteogenesis imperfecta, fractures, point-of-care ultrasound, emergency department

## Abstract

Patients with osteogenesis imperfecta (OI) are at an increased risk of pathological rib fractures even if there is no history of trauma. Early and accurate identification of such fractures are crucial for appropriate management. We present a case of a child with OI type 3 with multiple rib fractures who presented with transient cyanosis and increased work of breathing without a history of significant trauma. The patient’s chest radiographs were reported to have a single, new right posterior fourth rib fracture and an old, healing anterior fourth rib fracture. A point-of-care ultrasound performed by the attending pediatric emergency physician revealed additional findings of refracture over the old right anterior fourth rib fracture site and a new left posterior third rib fracture. These findings of multiple and bilateral rib fractures better account for the patient’s initial presentation. This case highlights the added advantages of ultrasound over conventional chest radiographs in the evaluation and diagnosis of a tachypnoeic pediatric patient with underlying metabolic bone disease and a complex skeletal structure with multiple pathological rib fractures but no chest tenderness.

## 1. Introduction

Rib fractures in the absence of a history of blunt trauma are extremely rare [1,2]. Osteogenesis imperfecta (OI) patients are at an increased risk for pathological fractures across all bones, and OI type 3 patients are at the greatest risk. Fractures in such patients might be minimally symptomatic and only found incidentally [3]. We present a case of an 8-year-old child with OI type 3 who was diagnosed with multiple pathological rib fractures using point-of-care ultrasound (POCUS); conventional radiographs reported only a single, new fracture and a preexisting, healing rib fracture.

## 2. Case Presentation

An 8-year-old child with a significant history of OI type 3 presented to the pediatric emergency department (PED) with a sudden onset cyanotic episode. The patient was diagnosed with OI type 3 at birth, and the patient’s medical history included over 50 fractures predominantly involving the limbs. The patient’s latest radiograph of the humerus three months ago visualized an incidental (asymptomatic) right anterior fourth rib fracture. The patient is on twice yearly zoledronic acid infusions, with a Z-score of −3.7 (lumbar spine) on the patient’s latest bone mineral density scan.

At the time of the incident, the patient was sitting in a child seat mounted onto a bicycle, wearing a restraint over both shoulders and the waist. As the bicycle traversed a speed bump, the patient suddenly developed shortness of breath with facial and perioral cyanosis lasting 3 min. Upon arrival to the PED, the patient was alert and cheerful. However, the patient was noted to be mildly tachypnoeic with a respiratory rate of 32 breaths per minute but was not in respiratory distress. The patient’s vital signs were otherwise appropriate for the patient’s age with a normal saturation (pulse oximetry saturation of 96%) of room air. Although unchanged from baseline, a physical examination was significant and showed the patient’s extremely short stature (body mass index: 21.2 kg/m^2^; height: 80 cm; weight: 13.3 kg, all less than third percentile for age) and deformities of all four limbs. A respiratory examination was unremarkable with good air entry bilaterally and no adventitious lung sounds. There was no chest wall tenderness or reported pain noted.

**Figure 1:**
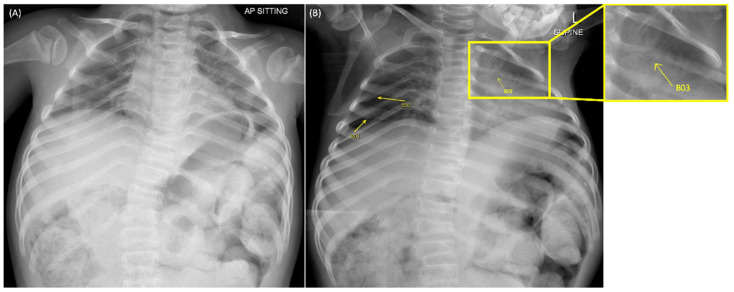
Chest radiographs of the patient; anterior−posterior chest radiograph (**A**) and left oblique chest radiograph (**B**). A right anterior fourth rib healing fracture with callus formation (B01) and a new right posterior fourth rib fracture (B02) were reported by the radiologists. There was a concern from the attending emergency physician about an unreported new fracture at the left posterior third rib (B03; enlarged view in yellow box).

In view of the patient’s comorbidities and high risk of fractures, chest radiographs (anterior−posterior and bilateral oblique), as shown in Figure 1A,B, were performed, alongside a capillary blood gas and echocardiograph (ECG). The capillary blood gas was normal, and the ECG showed normal sinus rhythm. The radiographs were formally reported as having only a single new fracture on the right posterior fourth rib and a healing fracture with callus formation on the right anterior fourth rib, which were previously observed on a radiograph a month earlier. However, the attending PED physician had concerns about another fracture over the left posterior third rib on the oblique chest radiograph (Figure 1B), which was not reported.

Due to the patient’s lack of chest wall tenderness and formal radiograph report indicating only a single (new) rib fracture, which may not account for the patient’s presentation, the attending PED physician proceeded to evaluate for occult pneumothorax and further rib fractures sonographically using a SonoSite M-Turbo ultrasound machine.

The POCUS examination did not reveal any pneumothorax. A rapid general screen for rib fractures was performed, followed by a more detailed ultrasound scan using longitudinal views along the ribs conducted with a microcurvilinear transducer at 5–8 MHz. The microcurvilinear transducer was selected due to the patient’s extremely small stature. In order to obtain the images, the patient was supine on the examination bed. The presence of disruptions on the hyperechoic line of the bony cortex was used to demonstrate sites of fractures [4].

**Figure 2:**
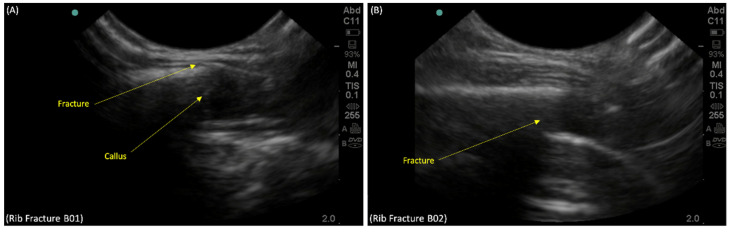
Point-of-care-ultrasound of the right fourth rib on longitudinal view showing: (**A**) a disruption in the callus of the old fracture on the right fourth anterior rib (B01), suggesting a refracture of the right fourth anterior rib and (**B**) cortical disruption (B02) of the right posterior fourth rib on longitudinal view using POCUS, suggesting a new fracture as reported in the chest radiographs.

An area of expanded cortex (callus) with focal discontinuity over the site of the old right anterior fourth rib fracture was observed, which represented a new undisplaced fracture through the callus (Figure 2A). This was not visualized or reported on chest radiography. The POCUS examination revealed the presence of the new right posterior fourth rib fracture, which can be seen as a focal disruption along the hyperechoic line of the bony cortex (Figure 2B).

**Figure 3:**
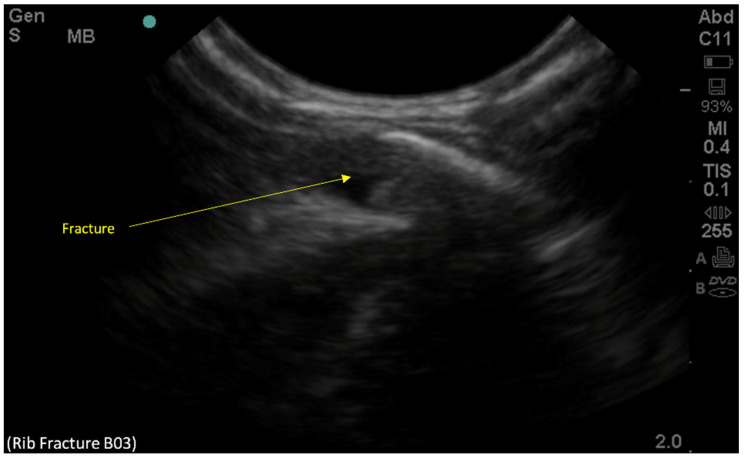
Point-of-care-ultrasound of the left posterior third rib on the longitudinal view demonstrating cortical disruption and suggesting a new fracture of the left posterior third rib, which was not reported in the chest radiographs.

At the site of contention, a clear discontinuity along the surface of the left posterior third rib proved the presence of an unreported fracture (Figure 3). The presence of these multiple rib fractures bilaterally could better account for the patient’s initial presentation.

After a consultation with the cardiothoracic surgery service for the multiple rib fractures, the child was admitted for closer monitoring. Further inpatient workup was performed, including an ultrasound of the patient’s diaphragm that showed normal excursion bilaterally. The patient was monitored as an inpatient and discharged the following day as the tachypnoea resolved.

## 3. Discussion

The current literature demonstrates that an US is not only a suitable alternative but is superior to radiography at delineating rib fractures. Numerous studies have shown that ultrasonography was able to detect rib fractures not seen in radiographs [5,6,7,8,9]. The reported sensitivity of POCUS to detect rib fractures in a systematic review was 97% with a specificity of 94% [9] while the sensitivity of chest radiography for detecting rib fractures was only 77% but had a higher specificity of 100%. However, this might be different in patients with metabolic bone diseases, such as osteogenesis imperfecta. There is limited published pediatric literature on supporting the role of ultrasound in evaluating patients for fractures when physical examination and conventional radiographs provide very limited clinical value. A review of the literature only found a single study by Kara et al. [7], who demonstrated that that US was able to detect cortical disruption and subperiosteal hematomas in 40% of asymptomatic patients with no visible fractures on radiographs. Similarly, we demonstrated in this case that US was able to detect an additional two rib fractures not seen on chest radiographs. There also remains a lack of evidence comparing the use of ultrasound and radiographs in delineating rib fractures in patients who have complicated skeletal anatomy, such as those with OI and other metabolic bone diseases.

Alongside the improved detection rates, the reported benefits of ultrasonography include decreased cumulative doses of ionising radiation, portability, repeatability, and time savings [5,6,7,8,9,10]. The reduction of ionizing radiation is particularly important with OI patients as these patients have been shown to have a small but significant increase in their lifetime cancer risk, especially in pediatric patients with such OI that would more likely warrant very frequent X-ray imaging [11,12].

While other imaging modalities have been proposed to detect pediatric rib fractures and, specifically, pathological ribs fractures, they are significantly limited by the high radiation exposure required in most of these modalities and lack cost effectiveness in the emergency department setting [13,14,15]. There have been reports of using chest computed tomography (CT) and microCT imaging to detect rib fractures in the pediatric population [13,14]. The significantly higher radiation exposure (compared to chest radiographs) and likelihood of frequent imaging in pediatric patients with OI type 3, who are likely to experience frequent and recurrent rib fractures, make CTs unsuitable as a first line imaging in the ED. However, if there are concerns of significant intrathoracic injuries, a chest CT may be of added value in this clinical context. Rib fractures, especially if subtle or noncostochondral, may not be accurately detected by magnetic resonance imaging [14,15]. The associated surrounding soft tissue swelling or bone marrow oedema seen in osseous fractures may be less well appreciated in pathological rib fractures in OI patients as there is often minimal or no trauma. Considerations of cost effectiveness, given the need for frequent imaging in this population in the emergency department setting, would further limit its use. Moreover, uncooperative pediatric patients may require sedation, and there may be potential risks with sedation in these patients due to potential musculoskeletal restrictive lung disease, and if multiple rib fractures are present, there can be risks of respiratory embarrassment. Similarly, the use of bone scintigraphy and positron emission tomography imaging, in addition to the radiation concerns listed above, may not be cost effective or feasible in the emergency department setting [14].

The use of ultrasound in the emergency department as the primary imaging modality or an imaging adjunct for diagnosing rib fractures in children with metabolic bone disorders and complex skeletal structures should be considered. More studies are required to better define its clinical role and validate the potential advantages of its use for detecting rib fractures in patients with OI and other metabolic bone diseases or congenital skeletal dysplasia in the emergency department.

## 4. Conclusions

We demonstrated that POCUS examination has increased utility in delineating rib fractures that were not seen on chest radiographs of a patient with a complex skeletal structure. This case report adds to the mounting evidence that supports the use of ultrasound to evaluate patients when there is no radiographic evidence but the clinical concerns for rib fractures remains. The potential added advantages of the use of POCUS for detection of pathological fractures in pediatric patients with osteogenesis imperfecta and other metabolic bone diseases should be further studied.

## Data Availability

Not applicable.
